# Self-Assembly of Discrete Porphyrin/Calix[4]tube Complexes Promoted by Potassium Ion Encapsulation

**DOI:** 10.3390/molecules26030704

**Published:** 2021-01-29

**Authors:** Massimiliano Gaeta, Elisabetta Rodolico, Maria E. Fragalà, Andrea Pappalardo, Ilenia Pisagatti, Giuseppe Gattuso, Anna Notti, Melchiorre F. Parisi, Roberto Purrello, Alessandro D’Urso

**Affiliations:** 1 Dipartimento di Scienze Chimiche, Università degli Studi di Catania, Viale A. Doria 6, 95125 Catania, Italy; gaetamassimiliano@libero.it (M.G.); elisabetta.rod27@gmail.com (E.R.); me.fragala@unict.it (M.E.F.); andrea.pappalardo@unict.it (A.P.); 2 Dipartimento di Scienze Chimiche, Biologiche, Farmaceutiche ed Ambientali, Università degli Studi di Messina, Viale F. Stagno d’Alcontres, 31, 98166 Messina, Italy; ipisagatti@unime.it (I.P.); ggattuso@unime.it (G.G.)

**Keywords:** noncovalent synthesis, hierarchical control, calixarenes, calix[4]tubes, metallo-porphyrins

## Abstract

The pivotal role played by potassium ions in the noncovalent synthesis of discrete porphyrin-calixarene nanostructures has been examined. The *flattened-cone* conformation adopted by the two cavities of octa-cationic calix[4]tube **C4T** was found to prevent the formation of complexes with well-defined stoichiometry between this novel water-soluble calixarene and the tetra-anionic phenylsulfonate porphyrin **CuTPPS**. Conversely, preorganization of **C4T** into a *C*_4v_-symmetrical scaffold, triggered by potassium ion encapsulation (**C4T**@K^+^), allowed us to carry out an efficient hierarchical self-assembly process leading to 2D and 3D nanostructures. The stepwise formation of discrete **CuTPPS/C4T**@K^+^ noncovalent assemblies, containing up to 33 molecular elements, was conveniently monitored by UV/vis spectroscopy by following the absorbance of the porphyrin Soret band.

## 1. Introduction

Porphyrins, owing to their redox [1,2] and opto-electronic properties [3,4,5], relative ease of derivatization [6] and propensity to self-organize in architectures of different size and topology [7,8,9], are very attractive building blocks for the synthesis of functional nanomaterials useful for light harvesting [10,11], sensing [12], catalysis [13], imaging [14] and photodynamic therapy [15] applications. Rods [16,17,18], wires [19,20,21], tubes [22], sheets [23], spheres [24] and rings [25] are examples of porphyrin-based nanostructure motifs reported to date.

In aqueous solution, however, one of the main obstacles to the development of discrete nanostructures is created by the pronounced tendency of porphyrins to spontaneously self-aggregate (via π–π stacking interactions), precluding the formation of arrays of well-defined shape and size. To overcome this, early endeavors have mainly focused on targeted covalent derivatizations or the formation of coordination bonds [26]. More recently, the rational design of porphyrin-based supramolecular assemblies has been successfully carried out in the presence of templating agents such as: polyelectrolites [27,28], peptides [29], inorganic molecules bearing metal-coordination centers [30,31,32,33] and macrocyclic compounds [34,35,36], by taking advantage of single or multiple metal coordination, hydrogen bonding, π–π stacking, electrostatic and hydrophobic interactions.

Multi-charged water-soluble calix[*n*]arenes [37,38,39,40], because of their remarkable affinity towards charge- and shape-complementary substrates [41,42,43,44], have been successfully employed as templating agents for the assembly of hybrid porphyrin-calixarene nanostructures in aqueous media [45,46] and in the solid state [47,48,49,50]. We have shown that both anionic [51] and cationic [52] calix[4]arenes quantitatively interact with oppositely charged porphyrins, under rigid hierarchical rules, providing assemblies with predictable sequence and stoichiometry. By replacing the calixarene framework with ditopic or tritopic bis- [53] or tris-calixarene [54] scaffolds we were able to control the dimensionality of the assembly, hierarchically forming 2D and 3D noncovalent architectures of considerable size. We were also able to induce chirality in some of these multi-component assemblies by using appropriate enantiopure agents [52,54,55]. The self-assembly in aqueous solution is mainly driven by electrostatic interactions between differently charged components as well as solvophobic effects and other noncovalent weak forces, all of which ultimately contribute to the thermodynamic stability of the species formed. The stability and kinetic inertness of these multicomponent assemblies have been assessed by light scattering, diffusion NMR studies [56] and a number of single-crystal XRD analyses [51,56].

**Scheme 1:**
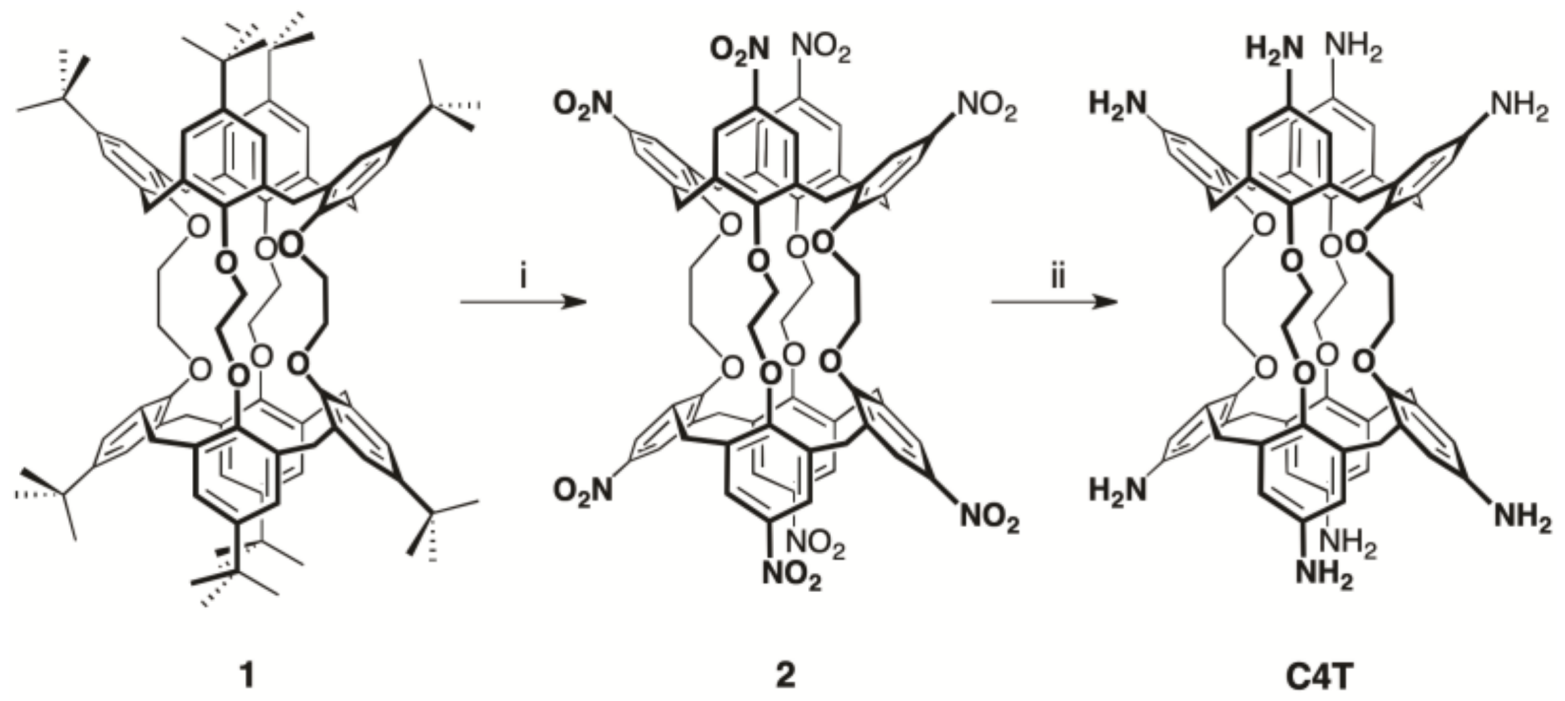
The synthesis of the octa-amino calix[4]tube C4T. Reaction conditions: (i) HNO_3_, CHCl_3_/AcOH, r.t., 72 h; (ii) H_2_, Raney/Ni, DMF, r.t., 24 h.

As a follow up to these studies, to test the limits of the noncovalent approach to the synthesis of porphyrin-calixarene nanostructures we have synthesized a water-soluble congener of the known *p-tert*-butylcalix[4]tube **1** (Scheme 1), first reported by Beer and coworkers [57], and now wish to report the profound effect played by a single potassium ion on the overall self-assembly process carried out in water in the presence of copper(II) *meso-tetrakis*-(4-sulfonatophenyl)porphyrin tetrasodium salt (**CuTPPS**). Unlike **CT4** (Scheme 1), the corresponding potassium complex (**C4T**@K^+^), because of the preorganization of its cavities and the rigidity of its ditopic tubular structure, is able to promote the noncovalent assembly of discrete porphyrin/calixtube nanostructures with a stoichiometry as high as 17:16.

## 2. Results and Discussion

Octa-amino calix[4]tube **C4T** was synthesized in overall 70% yield from *p-tert*-butylcalix[4]tube **1** [57] by exhaustive nitration followed by reduction of the resultant nitro derivative **2** (Scheme 1).

**Figure 1:**
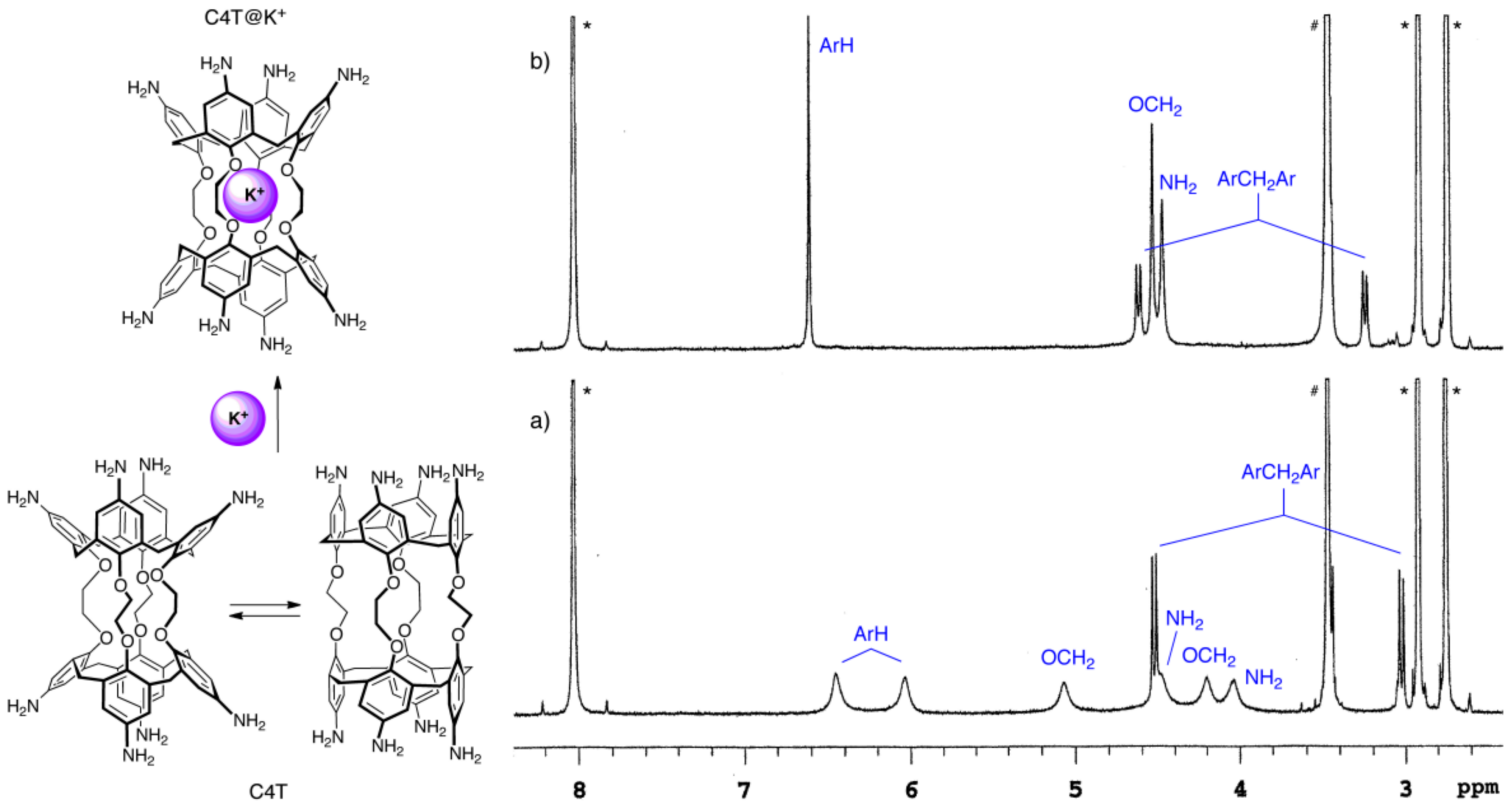
^1^H NMR (DMF-*d*_7_, 298 K) of: (**a**) octa-amino calix[4]tube **C4T**; (**b**) the potassium complex of the octa-amino calix[4]tube **C4T**@K^+^. The hashtag and the asterisks indicate the H_2_O and the residual solvent peaks, respectively.

Analogously to the parent *p-tert*-butylated derivative **1** [57] and a number of other calix[4]tubes [58,59,60,61,62], the two cavities of octa-amino calix[4]tube **C4T** adopt a preferential *flattened-cone* conformation (*C*_2v_ symmetry) in solution (DMF-*d*_7_, 298 K). Consistent with this, the ^1^H NMR spectrum displays pairs of equally intense broad singlets for the ArH, OCH_2_ and NH_2_ groups and a single AX-system for the ArCH_2_Ar bridging moieties (Figure 1a). Signal doubling is also seen in the ^13^C NMR spectrum of **C4T**.

**Chart 1:**
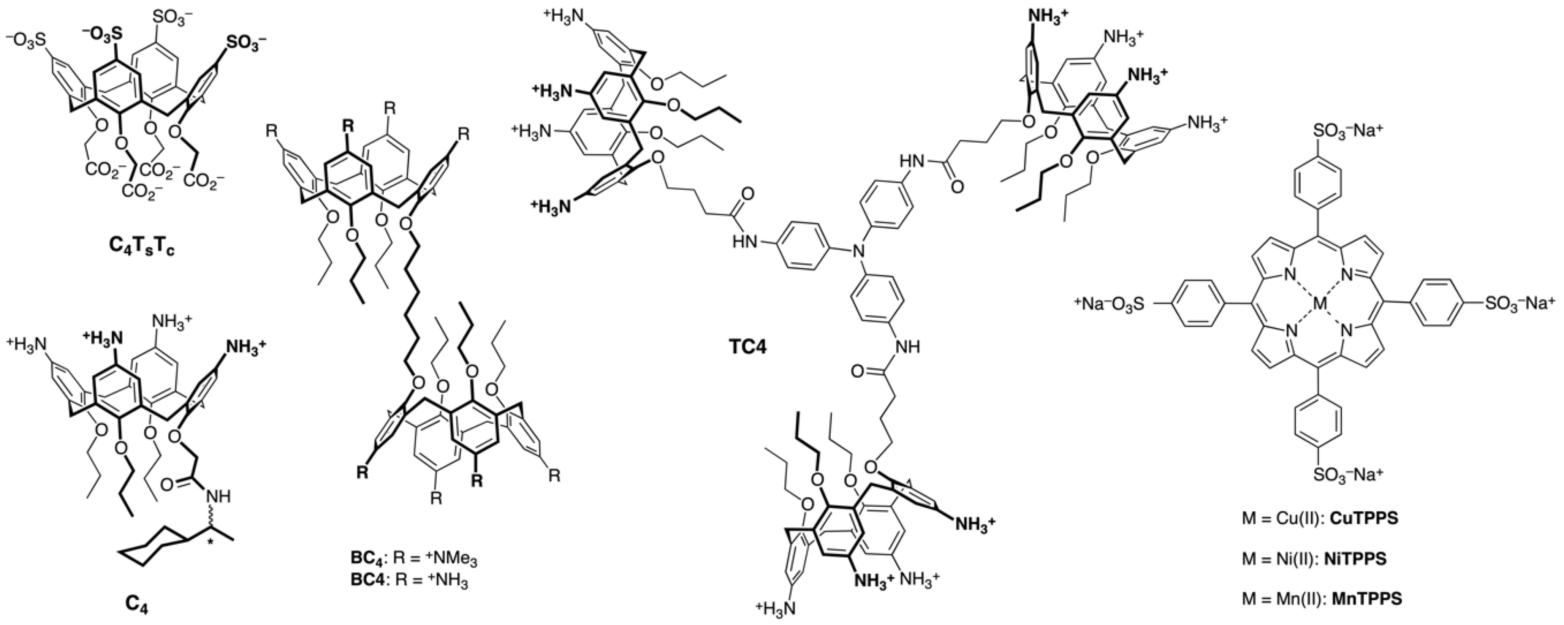
Calix[4]arene derivatives and metallo-porphyrins used in previous studies [51,52,53,54,55].

To assess the ability of calixtube **C4T** to act as a templating agent for the noncovalent synthesis of porphyrin-based supramolecular architectures, a 2.0 µM aqueous solution (pH = 3.0 [63]) of **C4T** was titrated with increasing aliquots of an aqueous solution of copper(II) *meso-tetrakis*-(4-sulfonatophenyl)porphyrin tetrasodium salt (**CuTPPS**). Earlier studies carried out on several water-soluble calix[4]arenes [51,52], bis-calix[4]arenes [52,55] and tris-calix[4]arenes [54], and a number of metallo-porphyrins (Chart 1), had shown that the formation of discrete calixarene/porphyrin assemblies proceeds in a step-wise hierarchical fashion [64] with the display of clear-cut spectral changes (absorption or emission). More specifically, the formation of any complex/assembly of well-defined stoichiometry is always pinpointed by a specific *break-point* on a diagram where the absorbance values of the porphyrin Soret band are plotted vs. the [porphyrin] × 4/[calixarene] ratio (*vide infra*). That is, an experimental data point of the titration curve where the slope variation is larger than 10%. Different slopes indicate the presence in solution of discrete assemblies, each of these characterized by a different molar extinction coefficient. In other words, the presence of *break-points* confirms that the species formed are not in equilibrium with each other, otherwise a straight line would only be observed over the entire course of a titration experiment.

**Figure 2:**
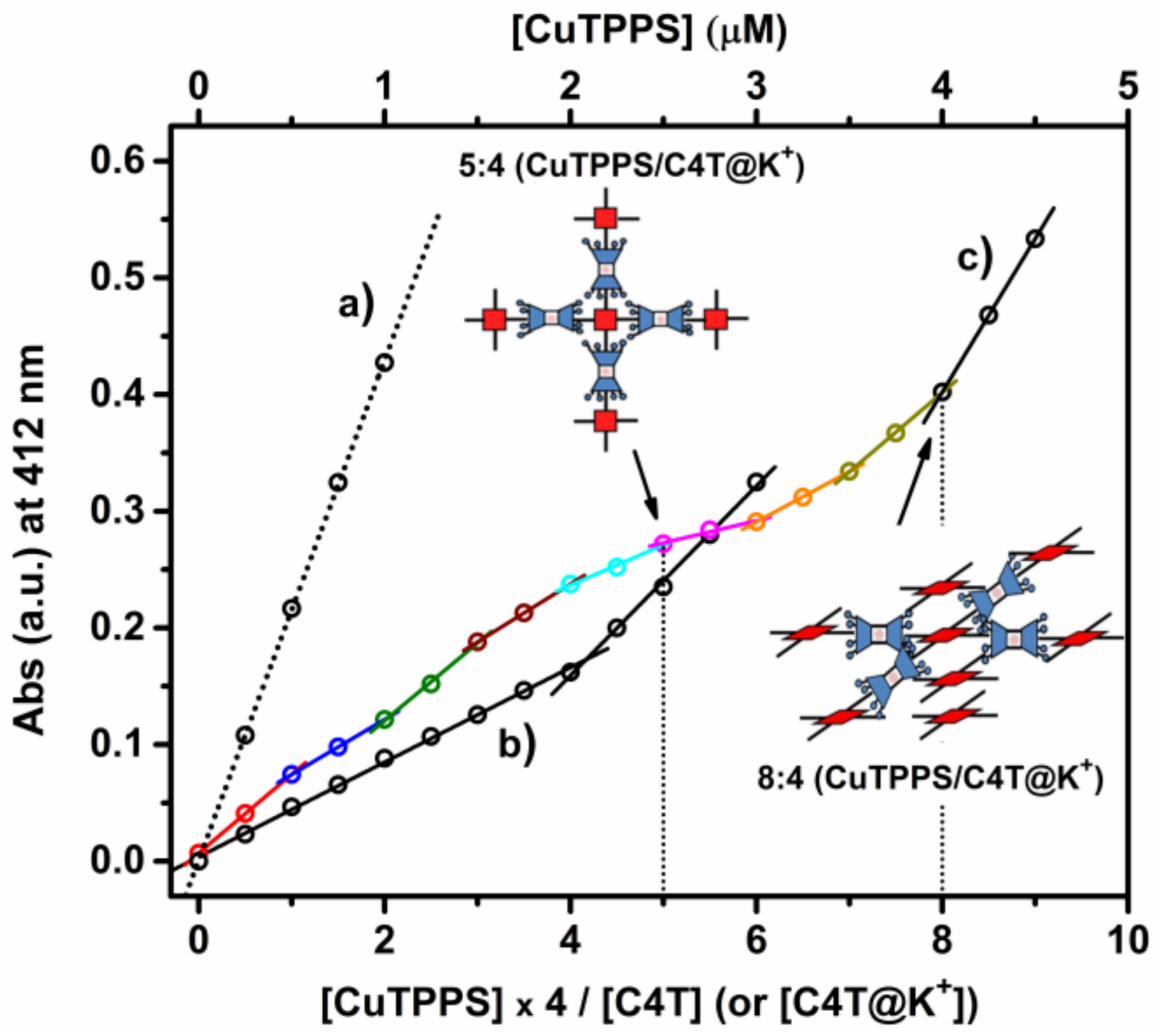
Variation in the absorbance of the CuTPPS Soret band (λ_max_ = 412 nm) observed upon: (i) increase of the porphyrin concentration in water (dotted black trace labelled as (**a**)); (ii) portion-wise addition of CuTPPS to a 2 μM aqueous solution of calix[4]tube C4T at pH = 3.0 (solid black trace labelled as (**b**)); (iii) portion-wise addition of CuTPPS to a 2 μM aqueous solution of C4T@K^+^ at pH = 3.0 (multicolored trace labelled as (**c**)).

In the case of the ditopic calix[4]tube **C4T** and the tetratopic porphyrin **CuTPPS**, however, no such trend was observed (Figure 2), despite the fact that the absorption data collected over the course of the titration displayed—with respect to the blank experiment carried out in the absence of **C4T**—hypochromicity and a broadening of the **CuTPPS** Soret band and, therefore, a clear indication of porphyrin–calixtube interactions (Figures S2 and S3, see the Supplementary Materials). The porphyrin–calix[4]tube assembly process was best analyzed by plotting the **CuTPPS** absorbance values at 412 nm vs. the [**CuTPPS**] × 4/[**C4T**] ratio. The trace joining the experimental data-points (Figure 2, trace b) is straight (i.e., no detectable *break-points*) up to the equivalence ([**CuTPPS**] = [**C4T**] = 2 µM) and then its slope becomes steeper upon further addition of **CuTPPS** (up to a 3 µM total concentration). This absorbance trend is consistent with an initial formation of **CuTPPS**/**C4T** complexes with a 1:1 stoichiometry, followed by a marked hyperchromic effect due to the absorbance of the porphyrin molecules present in excess in solution; the slope of the second segment of trace b approaches the one observed in **CuTPPS** solutions of increasing concentration (Figure 2, trace a). One possible explanation for this unexpected lack of stoichiometric complementarity between this oppositely charged pair is that the **C4T** cavities are forced to adopt a *flattened-cone* conformation by the four ethylene linkages (see above), resulting in poor preorganization. These structural features, because of steric hindrance and weaker electrostatic interactions, prevent the simultaneous binding of four calix[4]tube molecules to a single porphyrin and, as a result, the formation of a stable **CuTPPS**/**C4T** complex with a 1:4 stoichiometry and a cruciform structure (*vide infra*).

Given the known proclivity of *p-tert*-butylcalix[4]tube **1** to encapsulate potassium ions within the cryptand-like binding site, provided by the four dioxyethylene bridges, Beer’s findings [57,58] were exploited to “freeze” the cavities of the octa-amino calix[4]tube into a more favorable *cone* conformation. Accordingly, stirring of **C4T** in DMF in the presence of a large excess of KI, afforded the corresponding **C4T**@K^+^ potassium complex (Figure 1b).

To test whether the potassium complex of calix[4]tube **C4T** was able to promote supramolecular assembly as a result of its cavities being preorganized in a *C*_4v_-symmetry, the templating-agent potentials of **C4T**@K^+^ were reassessed under the conditions described earlier. The UV/vis titration was similarly carried out by adding increasing amounts of **CuTPPS** to a solution of **C4T**@K^+^ (2 µM) in water at pH = 3.0 [63]. As seen above, the spectra acquired in the course of the titration, showed that the absorbance of the porphyrin Soret band broadens and undergoes a hypochromic effect (Figure S4). However, compared with the **CuTPPS**/**C4T** system (Figure 2, trace b), in this case, the assembly process proceeds under stoichiometric control up to a [**CuTPPS**] = 4 µM, as unambiguously proven by the presence of several *break-points* coinciding with **CuTPPS**/**C4T**@K^+^ complexes of precise stoichiometry (i.e., 1:4-, 2:4-, 3:4-, 4:4-, 5:4-, 6:4-, 7:4- and 8:4-(**CuTPPS**/**C4T**@K^+^); see Figure 2, trace c). As the titration proceeds and further aliquots of porphyrin are added to the solution (i.e., for [**CuTPPS**] > 4 µM), the absorbance sharply increases and the related slope is seen to closely match the one detected for **CuTPPS** on its own (Figure 2, compare traces a with the last segment of trace c). These findings indicate that the excess porphyrin molecules now present in solution are no longer interacting with the supramolecular complex. As for the largest species obtained under these conditions, the absorption data of Figure 2 (trace c) suggests the formation of an 8:4-(**CuTPPS**/**C4T**@K^+^) supramolecular structure, similar to those observed elsewhere by single-crystal X-ray analysis [48,49,50,51], likely obtained as a result of the stacking of three additional porphyrin molecules above and/or below the plane containing the parent 5:4-(**CuTPPS**/**C4T**@K^+^) assembly [65], the latter having radially grown around the central **CuTPPS** unit in a step-wise and hierarchical fashion. Compared to the case analyzed at the beginning, where the two *C*_2v_-*symmetrical* cavities of **C4T** were seen to prevent efficient porphyrin binding, the result observed in the presence of the ditopic *C*_4v_-arranged **C4T**@K^+^ calixtube complex is quite dramatic in terms of stoichiometric control of the assembly process.

**Figure 3:**
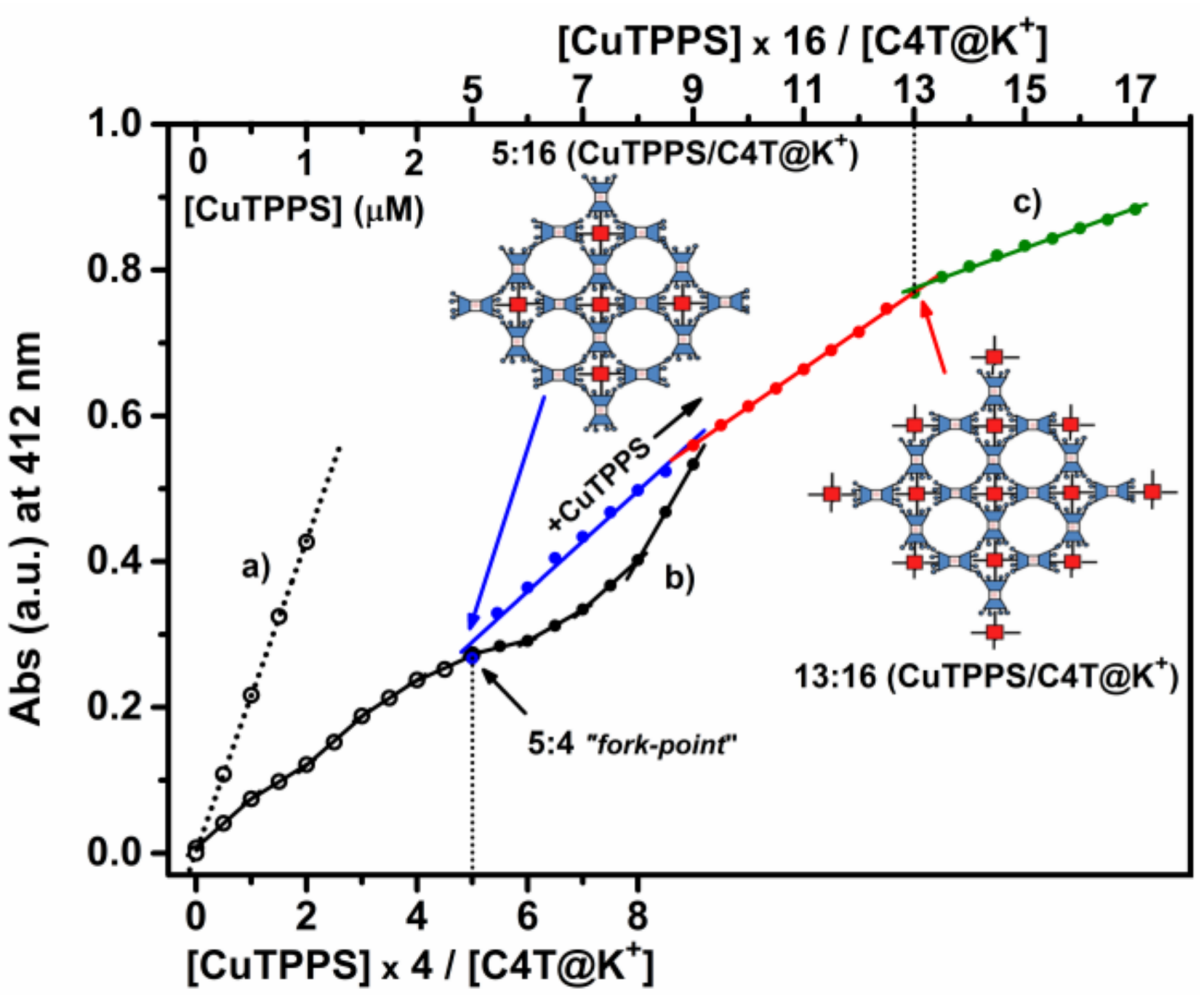
Variation in the absorbance of the **CuTPPS** Soret band (λ_max_ = 412 nm) observed upon: (i) increase of the porphyrin concentration in water (dotted black trace labelled as (**a**)); (ii) portion-wise addition of **CuTPPS** to a 2 μM aqueous solution of **C4T**@K^+^ at pH 3.0 (solid black trace labelled as (**b**)); (iii) initial portion-wise addition of **CuTPPS** to a 2 μM aqueous solution of **C4T**@K^+^ at pH 3.0 up to the formation of the 5:4-(**CuTPPS/C4T**@K^+^) assembly (see the black arrow of trace (**b**)), followed by portion-wise addition of **C4T**@K^+^ (up to a final [**C4T**@K^+^] = 8 μM) to form the 5:16-(**CuTPPS/C4T**@K^+^) assembly (see the blue arrow of trace (**c**)) and then final increment of the **CuTPPS** concentration from 2.5 to 8.5 μM to yield the 17:16-(**CuTPPS/C4T**@K^+^) assembly (multicolored portion of trace (**c**)).

Similar to the case of bis-calix[4]arene **BC_4_** (Chart 1, [53]), the 5:4-(**CuTPPS**/**C4T**@K^+^) assembly is a “fork-point” precursor, key to the subsequent 2D or 3D syntheses of larger porphyrin/calixtube supramolecular architectures (Figure 3).

Addition of porphyrins leads to the above-mentioned 8:4-(**CuTPPS**/**C4T**@K^+^) species, where the extra **CuTPPS** molecules are stacked above and/or below the planar 5:4-(**CuTPPS**/**C4T**@K^+^) assembly (second section of trace b), whereas an increase in the calixtube concentration (in a parallel titration experiment) from 2 to 8 µM prompts a planar growth of the assembly, yielding the 5:16-(**CuTPPS**/**C4T**@K^+^) species (Figure 3). As expected, given the absence of ‘free’ porphyrin molecules in solution, the formation of the latter proceeds with no variations in the absorbance at 412 nm, (blue arrow close to trace c in Figure 3, see also Figure S5). On the other hand, once the 5:16-(**CuTPPS**/**C4T**@K^+^) species has formed, a total of twelve cavities of calix[4]tube become available. As a result, upon subsequent increase of the **CuTPPS** concentration from 2.5 to 8.5 µM the solution undergoes—with respect to the blank, see trace a—a noticeable hypochromic effect (Figure 3, blue and red segments of trace c) which is consistent with the formation of an assembly with a 13:16 **CuTPPS**/**C4T**@K^+^ stoichiometry (Figure 3, red arrow close to trace c). In agreement with the even larger hypochromicity observed in the green segment of trace *c* (Figure 3), the formation of a new discrete species (i.e., 17:16-(**CuTPPS**/**C4T**@K^+^) is finally observed. This latter is probably formed by further piling of four **CuTPPS** molecules above and/or below the planar 13:16-(**CuTPPS**/**C4T**@K^+^) assembly. After reaching the 17:16 molar ratio the titration experiment was halted because of an incipient precipitate formation in solution.

## 3. Materials and Methods

### 3.1. General

Commercial reagent grade chemicals were used as received without any further purification. Solvents were dried by standard methods. Melting points were determined on a Kofler hot stage apparatus and are uncorrected. ^1^H and ^13^C NMR spectra were acquired at 25 °C in DMF-*d*_7_, at 500 and 125 MHz respectively. Chemical shifts are reported in ppm and are referenced to the solvent residual peak (δ_H_ = 2.75 ppm and δ_C_ = 29.8 ppm). The ESI-MS spectrum of **C4T**@K^+^ (dissolved in H_2_O at pH = 3.0) was recorded on an ES-MS Thermo-Finnigan LCQ-DECA instrument (positive ion mode) using a low declustering potential. UV/vis measurements were carried out at room temperature on a JASCO V-530 spectrophotometer. Quartz cuvettes with 1 cm path-length were used for all measurements. Routinely, 3 to 5 different solutions were used for each determination.

*p-tert*-Butylcalix[4]tube **1** was prepared according to a known literature procedure [57]. Copper(II) *meso-tetrakis*-(4-sulfonatophenyl)porphyrin tetrasodium salt (**CuTPPS**) was synthesized from the corresponding metal oxides (CuO) by heterogeneous metal-insertion in water, according to a previously reported procedure [65].

**CuTPPS, C4T** and **C4T**@K^+^ stock solutions (about 4 × 10^−4^, 3.2 × 10^−4^ and 5.4 × 10^−4^ M, respectively) were prepared in ultrapure water obtained from Elga Veolia Purelab flex and their concentrations were calculated spectrophotometrically (UV/vis in H_2_O) by looking at the maximum intensity of the porphyrin Soret band λ_max_(*ε*) = 412 nm (416,000 M^−1^ cm^−1^). **CuTPPS**/**C4T**@K^+^ assemblies were obtained at room temperature by adding increasing aliquots of **CuTPPS** (so that the concentration of the porphyrin in the titrating solution was 0.25 µM higher after each addition) to a 2 µM aqueous solution (pH = 3.0) of the **C4T**@K^+^ complex, up to desired molar ratio [**CuTPPS**]/[**C4T**@K^+^]. In water, under acidic conditions (pH = 3.0), the octa-amino calix[4]tube **C4T** is converted to its fully protonated octa-ammonium form(see Figure S1).

### 3.2. Syntheses of Calix[4]tube **C4T** and the Potassium Inclusion Complex **C4T**@K^+^

#### 3.2.1. Octa-Nitro Calix[4]tube **2**

Fuming HNO_3_ (4.5 mL) was slowly added to a cooled (T = −15 °C) solution of *p-tert*-butylcalix[4]tube **1** (630 mg, 0.45 mmol) in CHCl_3_ (180 mL) and AcOH (13.5 mL) and the mixture was then left to stir vigorously at room temperature. Addition of the same amounts of AcOH and HNO_3_ was repeated after 24 and 48 h. After 72 h, the solvent was evaporated under vacuum and the resulting residue was triturated with CH_3_OH (30 mL). Several recrystallizations from DMF of the solid thus obtained yielded derivative **2** as an off-white powder (554 mg, 94%). M.p. > 230 °C. ^1^H NMR (DMF-*d*_7_) δ 8.62 (s, ArH, 8H), 7.64 (s, ArH, 8H), 5.51 (s, OCH_2_, 8H), 4.85 and 4.03 (AX system, *J* = 13.6 Hz, ArCH_2_Ar, 16H), 4.84 (s, OCH_2_, 8H) ppm. ^13^C NMR (DMF-*d*_7_) δ 164.1, 162.1, 143.7, 143.1, 125.8, 124.5, 73.8, 73.4, 31.6 ppm. Anal. Calcd for C_64_H_48_N_8_O_24_: C, 58.54; H, 3.68; N, 8.53. Found: C, 58.23; H, 3.92; N, 8.37.

#### 3.2.2. Octa-Amino Calix[4]tube **C4T**

A suspension of **2** (100 mg, 0.076 mmol) and Raney/Nickel in DMF (50 mL) was stirred under H_2_ (1 atm) at room temperature for 24 h. The catalyst was filtered off and the eluate was evaporated to dryness under vacuum to provide a residue that upon treatment with MeOH gave **C4T** as a brown powder that was collected by suction filtration (61 mg, 75%). M.p. > 230 °C. ^1^H NMR (DMF-*d*_7_): δ 6.45 (br s, ArH, 8H), 6.03 (br s, ArH, 8H), 5.06 (br s, OCH_2_, 8H), 4.51 and 3.02 (AX system, *J* = 12.3 Hz, ArCH_2_Ar, 16 H), 4.47 (br s, NH_2_, 8H), 4.23 (br s, OCH_2_, 8H), 4.04 (br s, NH_2_, 8H) ppm. ^13^C NMR (DMF-*d*_7_): δ 150.6, 148.5, 143.3, 142.7, 135.9, 133.0, 115.07, 114.96, 73.7, 32.6 ppm. Anal. Calcd for C_64_H_64_N_8_O_8_: C, 71.62; H, 6.01, N, 10.44. Found: C, 71.31; H, 6.23; N, 10.29.

#### 3.2.3. Formation of the **C4T**@K^+^ Complex

A suspension of **C4T** (10 mg, 9 µmol) and KI (100 mg, 600 µmol) in dry DMF (30, mL) was kept under vigorous stirring at room temperature for 12 h. Excess of the inorganic salt was removed by suction filtration and the organic eluate was concentrated to dryness under vacuum to afford **C4T**@K^+^ as a solid residue. ^1^H NMR (DMF-*d*_7_): δ 6.60 (s, ArH, 16H), 4.63 and 3.26 (AX system, *J* = 13.8 Hz, ArCH_2_Ar, 16H), 4.53 (br s, OCH_2_, 16H), 4.48 (br s, NH_2_, 16H) ppm; ESI(+)-MS: *m/z* 287 ([M + K + 4H + Cl]^4+^, 17%).

## 4. Conclusions

Our findings show how minute structural modifications, of no apparent significance, may dramatically change the overall outcome of a self-assembly process, by either preventing or promoting the association of complex supramolecular nanostructures. Here, a single potassium ion is seen to produce an allosteric effect which triggers the subsequent noncovalent assembly of octa-cationic calix[4]tube **C4T** and the complementary tetra-anionic metallo-porphyrin **CuTPPS**. K^+^ binds to the cryptand-like binding site, formed by the four dioxoethylene bridging moieties of the calix[4]tube and, by acting as an “effector”, promotes the conformational change of its two cavities from *C*_2v_ to *C*_4v_. As a result, **C4T**@K^+^ is then able to act as an efficient templating agent, providing a variety of 2D and 3D **CuTPPS**/**C4T**@K^+^ assemblies of predictable sequence, topology and stoichiometry. The noncovalent synthesis of these species follows a strict hierarchical pattern which can, in principle, be exploited to introduce a given property (e.g., chirality [52]) at a specific location in the assembly so as to modulate the reactivity of the entire nanostructure.

We are currently investigating the ability of other ionic and neutral species to act as effectors of discrete noncovalent porphyrin-calixarene functional materials.

## Data Availability

Not available.

## Supplementary Materials

The following are available online, UV/Vis titration spectra, NMR and MS spectra.

## Sample Availability

Samples of the compounds **2** and **C4T** are available from the authors.
