# Supplementary material for: Self-Assembly of Discrete Porphyrin/Calix[4]tube Complexes Promoted by Potassium Ion Encapsulation
Source: Molecules. 2021 Jan 29;26(3):704. doi: 10.3390/molecules26030704 (PMC7866244; doi:10.3390/molecules26030704)
Supplement: Supplementary file 1 [file molecules-26-00704-s001.pdf]

SUPPLEMENTARY MATERIALS

Self-assembly of Discrete Porphyrin/Calix[4]tube Complexes Promoted by Potassium Ion Encapsulation

Massimiliano Gaeta [1], Elisabetta Rodolico [1], Maria E. Fragalà [1], Andrea Pappalardo [1], Ilenia Pisagatti [2], Giuseppe Gattuso [2], Anna Notti [2,*], Melchiorre F. Parisi [2,*], Roberto Purrello [1,*] and Alessandro D'Urso [1,*]

1 Dipartimento di Scienze Chimiche, Università degli Studi di Catania, Viale A. Doria 6, 95125 Catania, Italy; gaetamassimiliano@libero.it (M.G.); elisabetta.rod27@gmail.com (E.R.); me.fragala@unict.it (M.E.F.); andrea.pappalardo@unict.it (A.P.)

2 Dipartimento di Scienze Chimiche, Biologiche, Farmaceutiche ed Ambientali, Università degli Studi di Messina, Viale F. Stagno d'Alcontres, 31, 98166 Messina, Italy; ggattuso@unime.it (G.G.); ipisagatti@unime.it (I.P.)

* Correspondence: adurso@unict.it (A.D.); anotti@unime.it (A.N.); mparisi@unime.it (M.F.P.); rpurrello@unict.it (R.P.)

Table of Contents:

Figure S1. pH titration of **C4T**....2

Figure S2. UV/vis titration spectra of **CuTPPS** 3

Figure S3. UV/vis titration spectra of the **CuTPPS/C4T** complexes....3

Figure S4. UV/vis spectra of the **CuTPPS/C4T**@K^+ assemblies....4

Figure S5. UV/vis spectra of the 5:4- and 13:16-(**CuTPPS/C4T**@K^+) assemblies....4

Figure S6. ^{1}H NMR of octa-nitro calix[4]tube **2**....5

Figure S7. ^{13}C NMR of octa-nitro calix[4]tube **2**....5

Figure S8. ^{1}H NMR of octa-amino calix[4]tube **C4T**....**5**

Figure S9. ^{13}C NMR of octa-amino-calix[4]tube **C4T**....**5**

Figure S10. HMQC NMR of octa-amino calix[4]tube **C4T**....**6**

Figure S11. ESI-MS spectrum of **C4T@K^+**....**6**

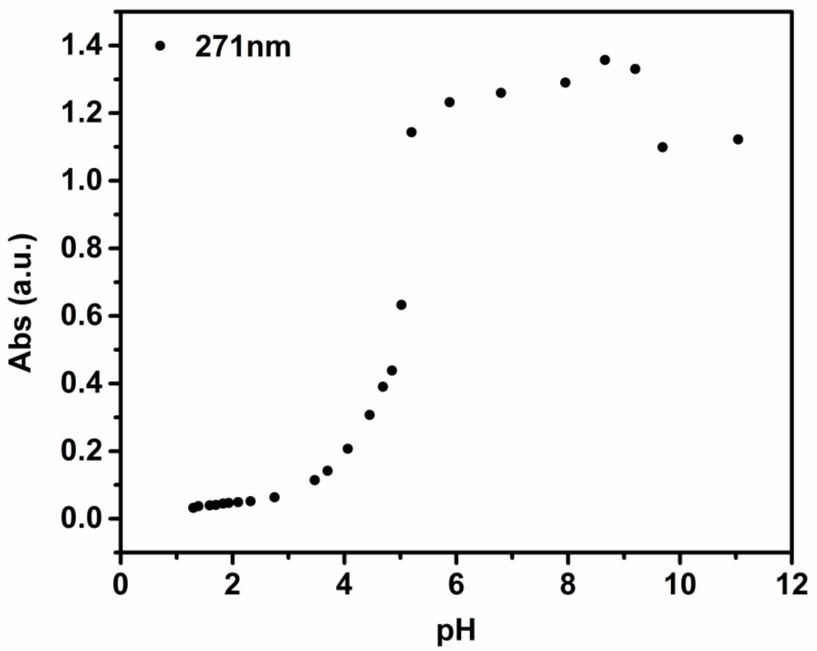

Figure S1. Absorbance variation (at 271 nm) *vs* pH of a 20 μM water solution (pH = 3.0) of octa-amino calix[4]tube **C4T**.

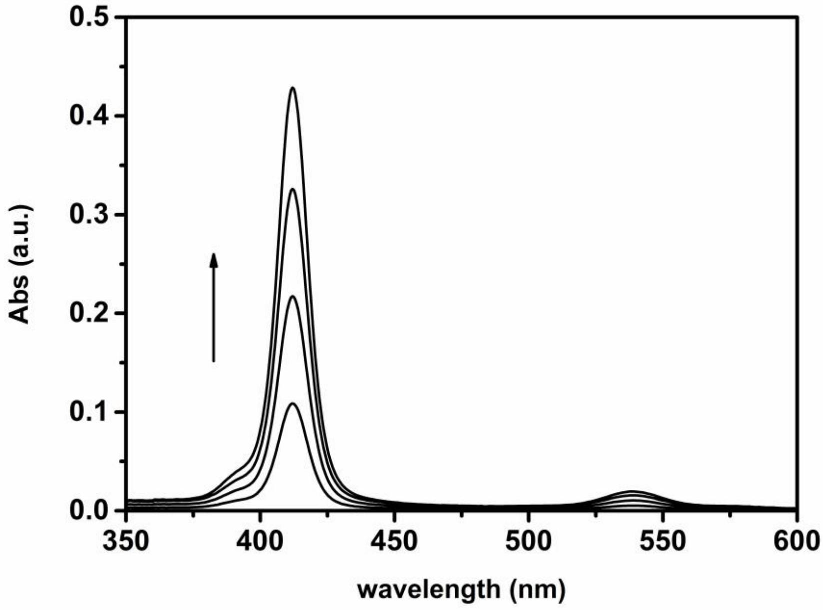

Figure S2. UV/vis absorption spectra of **CuTPPS** in aqueous solution at pH = 3.0 ([**CuTPPS**] ranged from 0.25 to 1 μM).

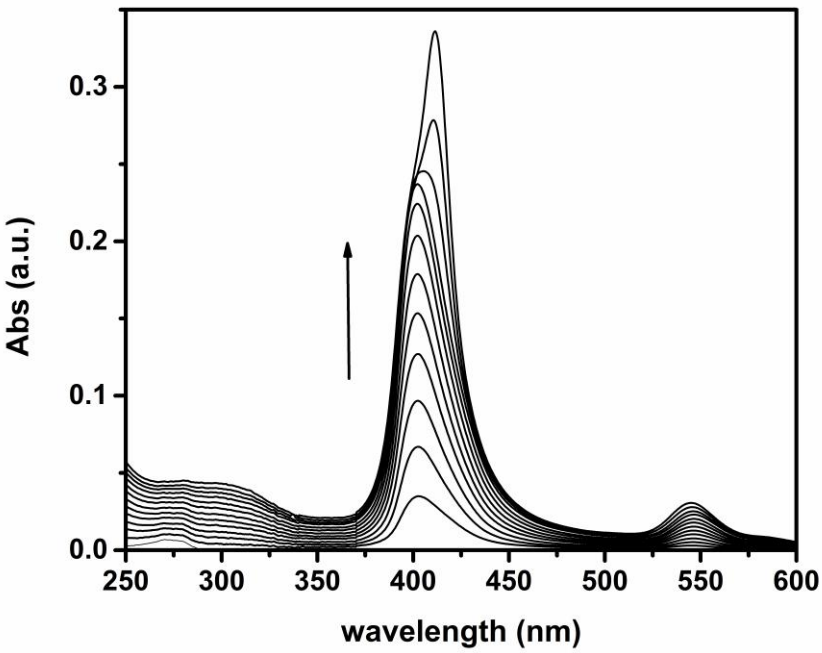

Figure S3. UV/vis absorption spectra recorded over the course of the titration of a 2 μM aqueous solution of **C4T** at pH 3.0 with successive aliquots of an aqueous solution of **CuTPPS** ([**CuTPPS**] ranged from 0.25 to 3.0 μM).

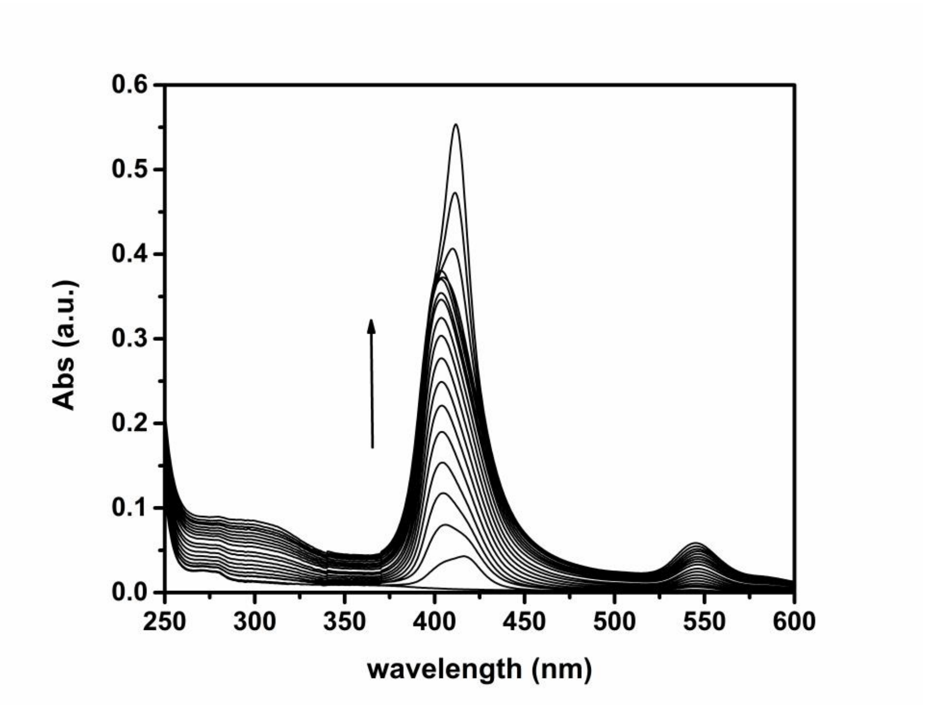

Figure S4. UV/vis absorption spectra recorded over the course of the titration of a 2 μM aqueous solution of **C4T**@K+ at pH = 3 with successive aliquots of an aqueous solution of **CuTPPS** ([**CuTPPS**] ranged from 0.25 to 4.5 μM).

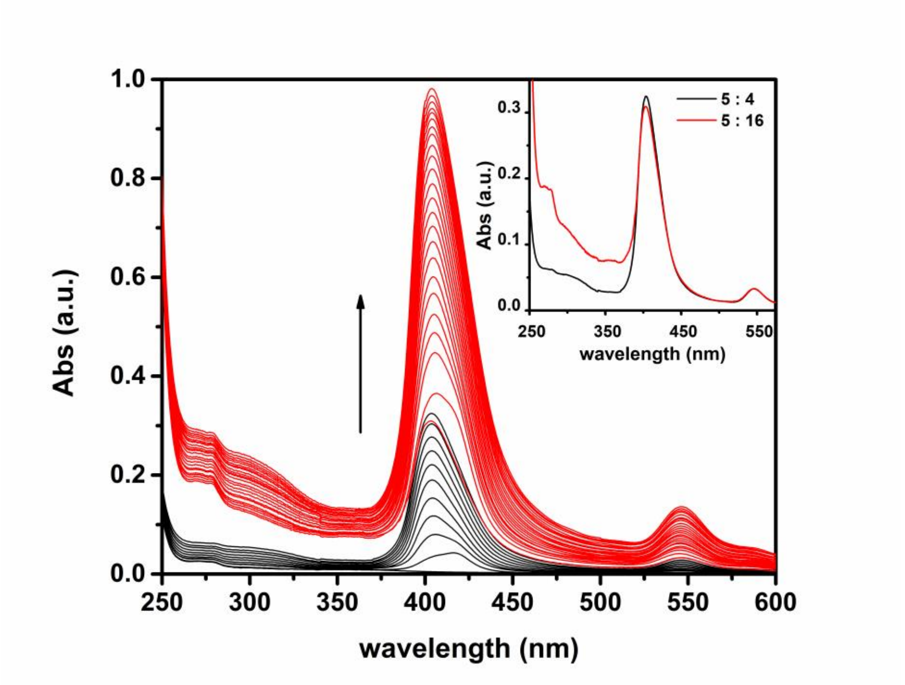

Figure S5. UV/vis absorption spectra (λ_{max} = 412 nm) recorded over the course of the titration of a 2 μM aqueous solution of **C4T**@K+ at pH = 3 with: *i)* successive aliquots of an aqueous solution of **CuTPPS** ([**CuTPPS**] ranged from 0.25 to 2.5 μM) (black traces) and *ii)* successive aliquots of **CuTPPS** ([**CuTPPS**] ranged from 2.5 to 8.5 μM) after increasing the concentration of **C4T**@K+ to 8 μM. The inset shows the spectra of the 5:4-(**CuTPPS/C4T**@K+) and the 5:16-(**CuTPPS/C4T**@K+) assemblies (black and red traces respectively).

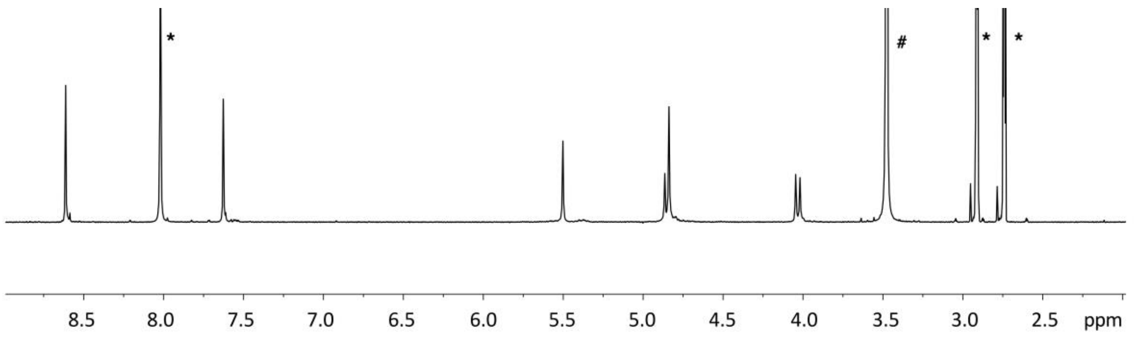

Figure S6. ^{1}H NMR (DMF-d_7, 298 K) of octa-nitro calix[4]tube **2**. The hashtag and the asterisks indicate the H_2O and the residual solvent peaks respectively.

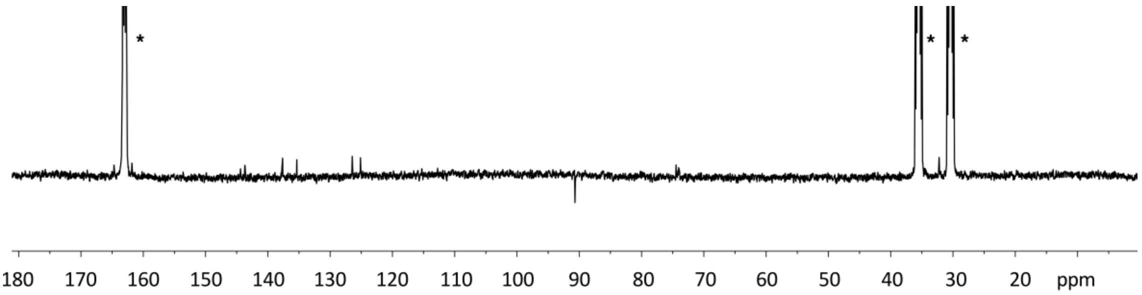

Figure S7. ^{13}C NMR (DMF-d_7, 298 K) of octa nitro calix[4]tube **2**. Asterisks indicate the residual solvent peaks.

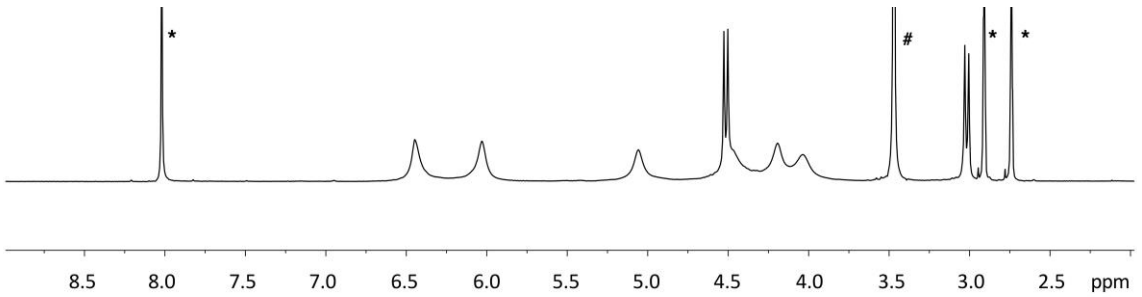

Figure S8. ^{1}H NMR (DMF-d_7, 298 K) of octa-amino calix[4]tube **C4T**. The hashtag and the asterisks indicate the H_2O and the residual solvent peaks respectively.

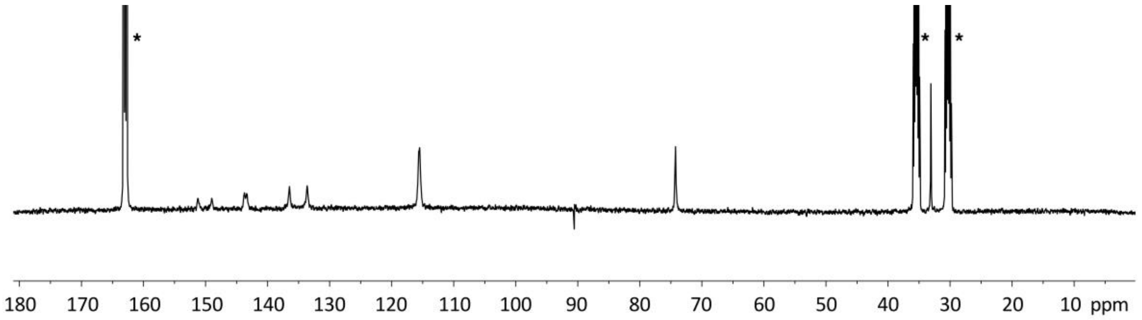

Figure S9. ^{13}C NMR (DMF-d_7, 298 K) of octa-amino calix[4]tube **C4T**. Asterisks indicate the residual solvent peaks.

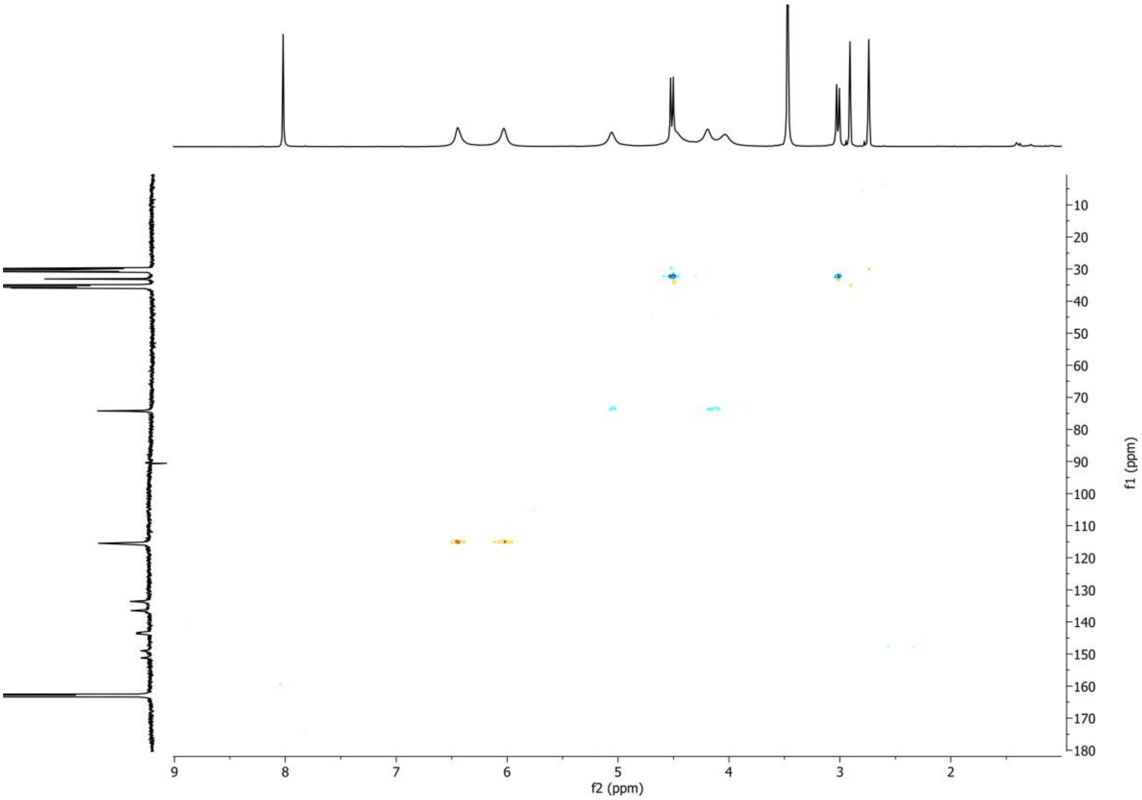

Figure S10. HMQC NMR (DMF-*d*7, 298 K) of octa-amino calix[4]tube **C4T**.

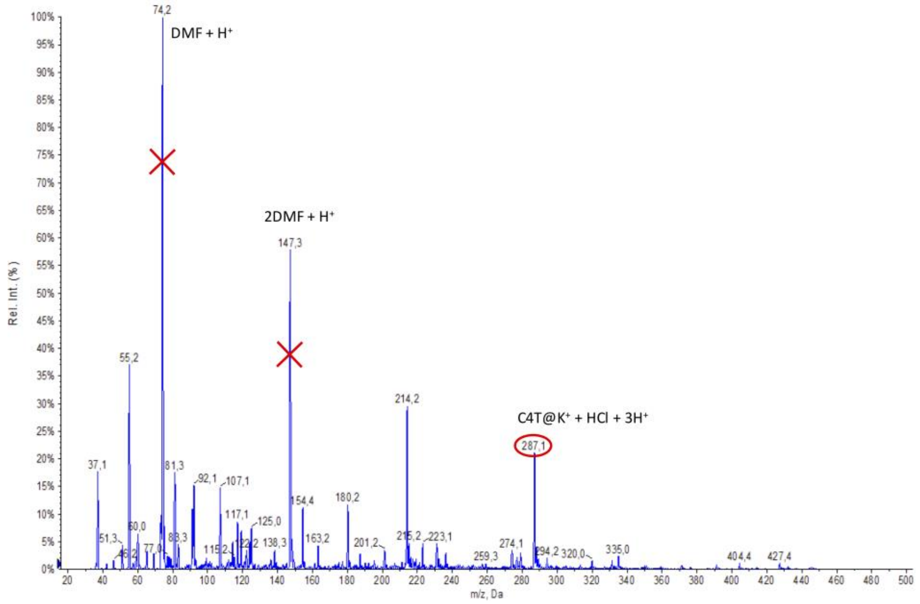

Figure S11. ESI(+)-MS spectrum of the potassium complex of octa-ammonium calix[4]tube **C4T**@K+.
